# Post-translational modifications are enriched within protein functional groups important to bacterial adaptation within a deep-sea hydrothermal vent environment

**DOI:** 10.1186/s40168-016-0194-x

**Published:** 2016-09-06

**Authors:** Weipeng Zhang, Jin Sun, Huiluo Cao, Renmao Tian, Lin Cai, Wei Ding, Pei-Yuan Qian

**Affiliations:** Division of Life Science, Hong Kong University of Science and Technology, Clear Water Bay, Hong Kong

**Keywords:** Hydrothermal vent, Post-translational modification, Nitrospirae, Metaproteomics

## Abstract

**Background:**

Post-translational modification (PTM) of proteins is one important strategy employed by bacteria for environmental adaptation. However, PTM profiles in deep-sea microbes remain largely unexplored.

**Results:**

We provide here insight into PTMs in a hydrothermal vent microbial community through integration of metagenomics and metaproteomics. In total, 2919 unique proteins and 1306 unique PTMs were identified, whereas the latter included acetylation, deamination, hydroxylation, methylation, nitrosylation, oxidation, and phosphorylation. These modifications were unevenly distributed among microbial taxonomic and functional categories. A connection between modification types and particular functions was demonstrated. Interestingly, PTMs differed among the orthologous proteins derived from different bacterial groups. Furthermore, proteomic mapping to the draft genome of a Nitrospirae bacterium revealed novel modifications for proteins that participate in energy metabolism, signal transduction, and inorganic ion transport.

**Conclusions:**

Our results suggest that PTMs are enriched in specific functions, which would be important for microbial adaptation to extreme conditions of the hydrothermal vent. PTMs in deep-sea are highly diverse and divergent, and much broader investigations are needed to obtain a better understanding of their functional roles.

**Electronic supplementary material:**

The online version of this article (doi:10.1186/s40168-016-0194-x) contains supplementary material, which is available to authorized users.

## Background

Hydrothermal vents are cracks in the earth’s crust where high-temperature water escapes after being heated in the below rocks. Scientists have explored the deep ocean hydrothermal vents and were surprised to find the areas teeming with abundant life [1–3]. As important players, microbial populations participate in diverse biogeochemical processes, including the nitrogen, sulfur, and carbon cycles. Microbes in hydrothermal fields are mostly sustained by energy derived from inorganic redox reactions. Despite the common general origin of the investigated hydrothermal vents and the important roles of microbial communities, mechanisms underlying microbial adaptation to the vent environments remain largely unknown.

As one of the important strategies for environmental adaptation, post-translational modifications (PTMs) play crucial roles in regulating protein function and controlling several fundamental features of microbial biology, such as cell signaling, protein turnover, cell-cell interactions, and cell differentiation. For example, protein methylation denotes the addition of a methyl group to a protein or the substitution of an atom or group by a methyl group, and it is involved in mediating protein-protein interactions and enhancing protein thermostability [4]; the hydroxylation of specific residues in the ribosome has been identified in bacteria, suggesting a role for hydroxylation in cell growth and cycling [5]; in addition, phosphorylation and methylation collectively regulate signal transduction in bacteria [6, 7].

Among the studies focused on PTMs to date, laboratory strains have served as models or research subjects in most cases. However, the microbial community in nature is very complex, and thus, characterizing PTM events in natural communities is a challenging task. Li et al. identified PTMs in two growth stages of acid mine drainage (AMD) biofilms using a shortgun proteomics approach. They analyzed the PTMs profile based on an enrichment-independent technique that allowed the direct quantification of different modification events and characterized eight common biological PTM types [8]. More recently, Marlow et al. studied protein PTMs in microbial communities from marine methane seep habitats and focused on PTMs of methyl-coenzyme M reductase (Mcr) orthologs [9]. These studies have provided insights into PTMs in natural microbial communities, which are barely being explored.

In the present study, we employed metaproteomics, metagenomics, and genome binning to explore the PTMs in the microbial community from a hydrothermal vent plume on the South Mid Atlantic Ridge (SMAR). The findings have provided new insight into PTM events in deep-sea extreme areas and motivated further study of their roles in microbial ecology and physiology.

## Results

### Overview of the metagenome, metaproteome, and PTMs

Information for the assembled metagenome is summarized in Additional file 1: Table S1. The total number of contigs was 24,099, with an N50 of 10,361 bp, to generate 171,515 open reading frames (ORFs). These ORFs were used as a database for the following metaproteomic analysis. Using gel-based fractionation (eight fractions for each metaproteomic sample) of the free-labeled peptides and LTQ-Orbitrap-MS/MS analysis (work flow shown in Additional file 1: Figure S1), we identified in total 2919 unique proteins, from 1,978,700 peptide-spectrum matches for the two metaproteomes (metaproteo-1 and metaproteo-2). Among these proteins, 766 were shared by the two metaproteomes. The high-resolution MS/MS generated a high mass accuracy (<0.02 Da mass error and a very low false discovery rate of 0.1 %). Mascot Daemon searching produced a total of 1306 unique PTMs for the two metaproteomes (PTM1 and PTM2), including acetylation, deamination, hydroxylation, methylation, nitrosylation, oxidation, and phosphorylation. Detailed information regarding the number of identified proteins and PTMs in the two metaproteomes is summarized in Additional file 1: Figure S2, and all PTMs are listed in Additional file 2.

### Taxonomic distribution of the metagenome, metaproteome, and PTMs

**Fig. 1:**
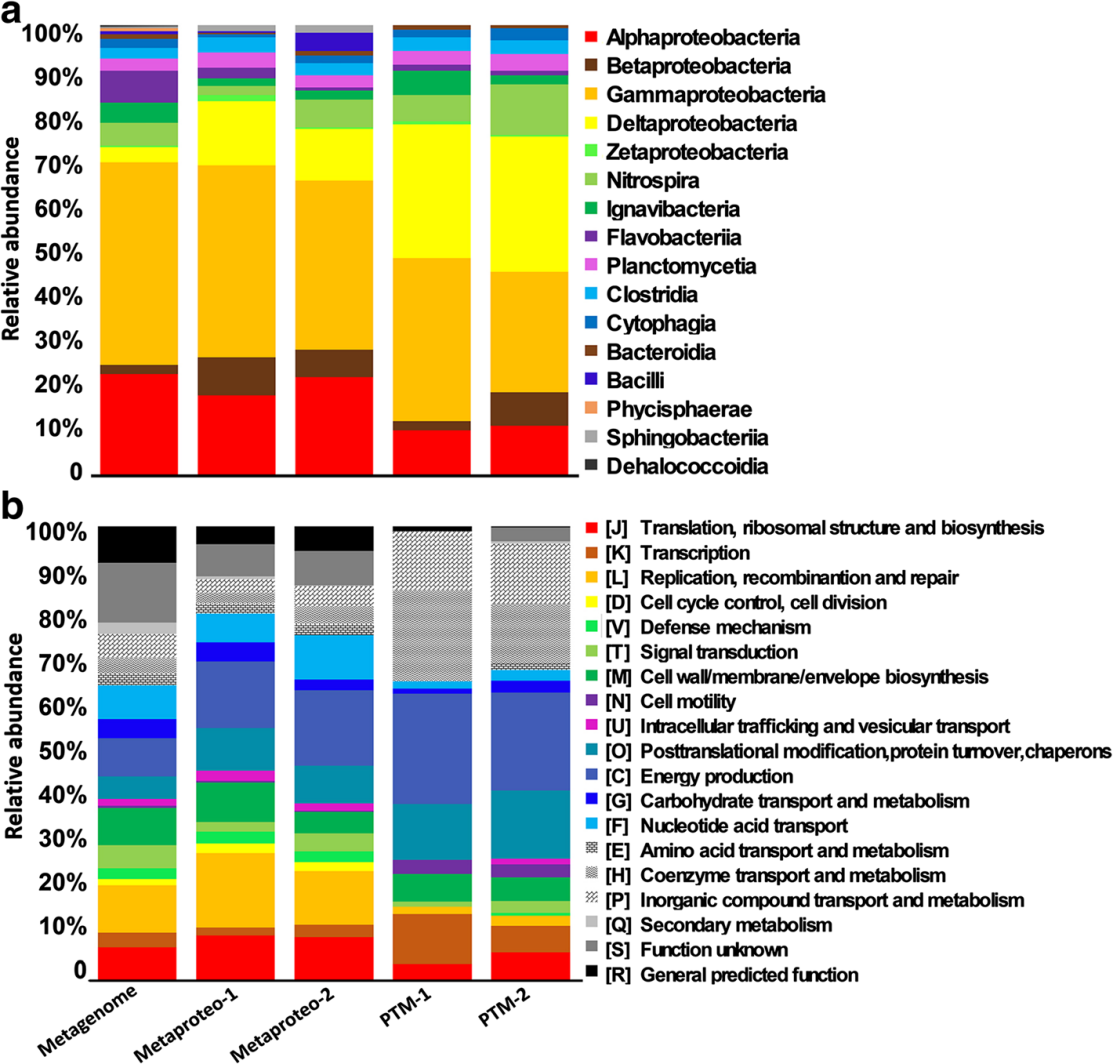
Taxonomic and functional structures of the metagenome, metaproteome, and PTMs. One metagenomic and two metaproteomic samples were included in the analysis. **a** Taxonomic classification at the class level based on conserved single-copy proteins. **b** Functional classification according to the COG categories

The hidden Markov models (HMMs) of conserved single-copy proteins were extracted from the metagenome and metaproteome-derived ORFs and searched against the National Center for Biotechnology Information (NCBI)-Nr database to reveal the taxonomic structures. Assignment of the reads at the class level revealed the prevalence of Alphaproteobacteria, Betaproteobacteria, Gammaptoteobacteria, Deltaptoteobacteria, and Nitrospira (phylum Nitrospirae) (Fig. 1a). Compared with the metagenome, the metaproteomes were enriched for Deltaptoteobacteria, which were clearly more enriched in the PTM profiles (Student’s *t* test, *P* < 0.005, PTMs versus metaproteomes). Moreover, bacteria belonging to the phylum Nitrospirae were also enriched in the PTM profiles (*P* < 0.05, PTMs versus metaproteomes).

### Functional distribution of the metagenome, metaproteome, and PTMs

All of the ORFs from the metagenome and metaproteomes were searched against the protein databases, including Clusters of Orthologous Groups (COGs), Kyoto Encyclopedia of Genes and Genomes (KEGG), and NCBI-Nr, to identify the functional profiles. The distribution of COG functional categories is summarized in Fig. 1b. One of the notable results was that the genes responsible for translation [J] and replication [L] accounted for 20–30 % of the metagenome and metaproteome, but their total abundance decreased to <7 % in the PTMs. In contrast, several COG functional categories were significantly enriched for PTMs when compared with the metaproteomes, including transcription [K] (Student’s *t* test, *P* < 0.05) cell motility [N] (*P* < 0.05), energy production [C] (*P* < 0.05), coenzyme transport and metabolism [H] (*P* < 0.01), and inorganic ion transport and metabolism [P] (*P* < 0.05).

**Fig. 2:**
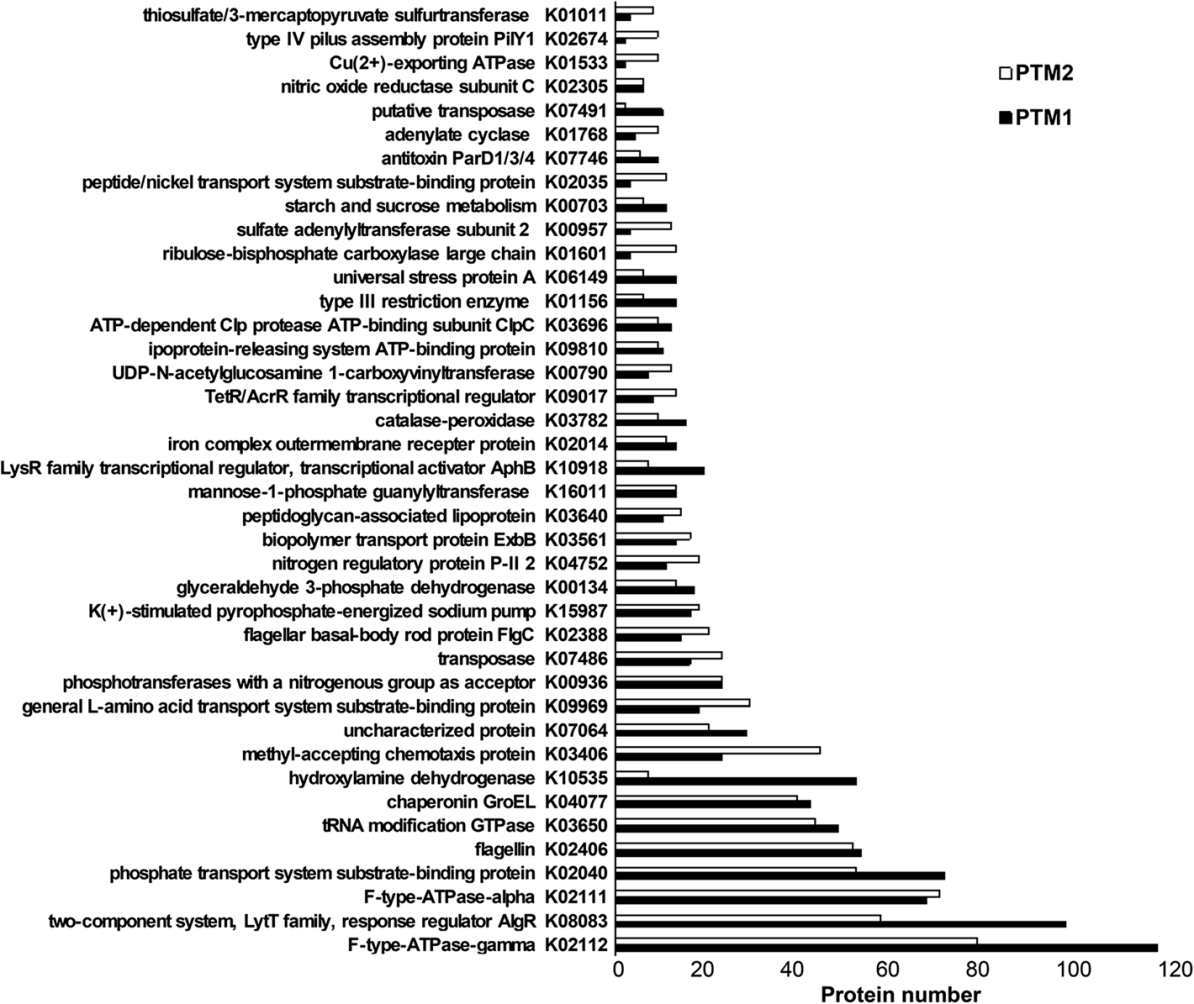
The 40 proteins with the most abundant PTMs. The proteins were annotated according to the KEGG database. The results derived from the two metaproteomes are shown, which were designated as PTM1 and PTM2, respectively

To confirm the functional profiles, individual genes annotated by KEGG are summarized in Fig. 2. The most abundant proteins with modifications included those related to electron transport and energy production, such as F-type ATPase and ribulose-biophosphate carboxylase; inorganic ion metabolism, such as nitric oxide reductase, phosphate transport system protein, iron complex outer membrane receptor protein, sulfate adenylyltransferase, and Cu2^+^-exporting ATPase; and signal transduction and chemotaxis, such as TetR and AcrR family transcriptional regulators and FlgC. The prevalence of these genes was consistent with the COG categories. In addition, a number of genes responsible for gene recombination, such as transposase and restriction enzymes, were identified.

### Correlation between PTM types and functional categories

**Fig. 3:**
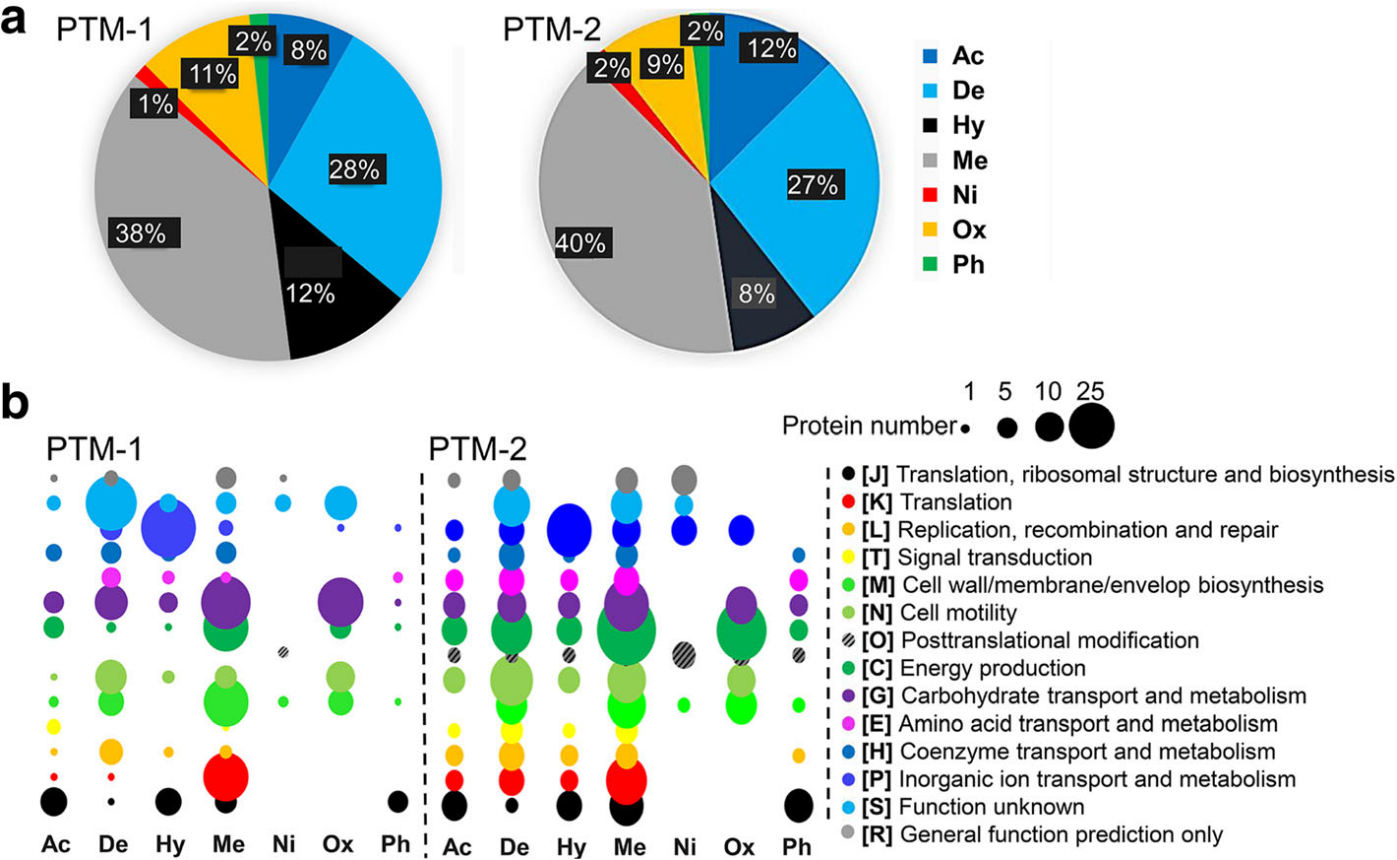
Correlation between PTM types and functional categories. **a** Percentages of the seven different PTM types. **b** Distribution of the seven PTM types among the COG categories. *Ac* acetylation, *De* deamination, *Hy* hydroxylation, *Me* methylation, *Ni* nitrosylation, *Ox* oxidation, *Ph* phosphorylation

The above results revealed the enrichment of PTMs in particular pathways, such as inorganic ion transport and energy metabolism. It can be hypothesized that the distribution of PTM types in different functional categories was also uneven because molecular studies of model species have demonstrated that divergent modifications exert distinct functions. Thus, we first summarized the percentages of the seven different PTM types (Fig. 3a). Methylation was the most prevalent PTM type, accounting for 40 % of the PTMs, followed by deamination, which accounted for 30 %. In contrast, nitrosylation and phosphorylation only accounted for 1–2 %. We distributed the seven PTM types into COG categories, as shown in Fig. 3b. Proteins that are important for inorganic ion metabolism [P] were mainly associated with hydroxylation; energy production [C] was associated with methylation, oxidation, and deamination; and cell motility was associated with deamination and methylation.

We further explored the PTMs in orthologous proteins from different taxa. Examples included F-type ATPases belonging to Bacteroidia, Gammaproteobacteria, Flavobacteriia, and Alphaproteobacteria based on the MEtaGenome ANalyzer (MEGAN) analysis (Additional file 1: Figure S3). The results revealed the divergence of PTMs, although the selected amino acid sequences were rather conserved. The modification types included acetylation, deamination, methylation, and oxidation, whereas the modification sites included lysine, glutamine, and methionine.

### Genome information for the dominant microbe, Nitrospirae bacterium sp. nov

To further understand the organization of functions and PTMs, we recovered one draft genome from the metagenome dataset. Based on the phylogenetic tree constructed using 16S ribosomal RNA (rRNA) genes (Additional file 1: Figure S4), the bacterium had a close phylogenetic relationship with the bacterial members of the phylum Nitrospirae. The bacterium was located in the same branch as uncultured bacteria from hydrothermal vent areas, which may have adapted to such extreme environments for a long time but for which no genome information is available. The bacterium was named Nitrospirae bacterium sp. nov. Phylogeny of single-copy genes derived from available Nitrospirae genomes and Nitrospirae bacterium sp. nov was also investigated (Additional file 1: Figure S5), which placed this new Nitrospirae bacterium at a similar location as that of the 16S rRNA gene tree. The completeness of the genome of Nitrospirae sp. nov was estimated based on the number of the 139 conserved single-copy protein-encoding genes and by comparison with previously reported Nitrospirae genomes that served as references (Additional file 1: Table S2). The results showed that 20 genes, such as GAD domain-containing protein (PF02938), potentially could not be recovered from the genome bin of Nitrospirae bacterium sp. nov because these genes were present in the reference genomes. In contrast, the presence of two elongation factor TS (PF00889) and two pseudouridine synthase I (PF01416) in the reference genomes indicated that the observed duplication in the genome of Nitrospirae bacterium sp. nov was not due to contamination. Thus, we assumed that the completeness of the draft genomes was 85.6 %. Contigs of the Nitrospirae bacterium sp. nov genome were further compared with the reference genomes, which revealed that it shared 79.8 % of the genome inventory with other Nitrospirae bacteria.

### Metabolic pathways and PTMs in Nitrospirae bacterium sp. nov

**Fig. 4:**
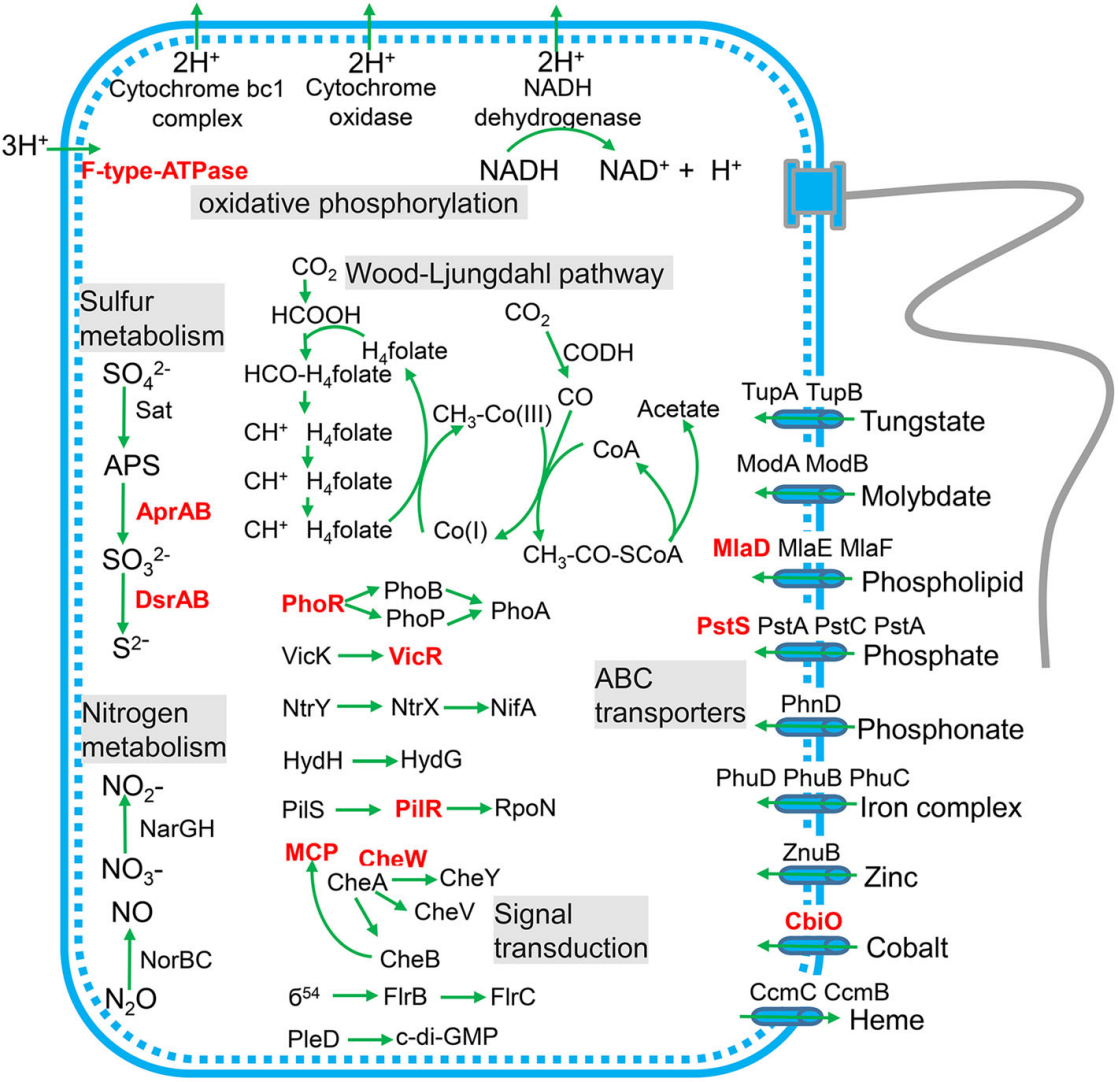
The metabolic capacities and pathways with enriched PTMs of Nitrospirae bacterium sp. nov. This bacterium possesses multiple pathways for energy metabolism, signal transduction, and inorganic ion transport, which contain several proteins with PTMs (highlighted in *red*)

Catalytic pathways were constructed based on the genome of Nitrospira bacterium sp. nov by blastp searches against the KEGG database (Fig. 4). The Wood-Ljungdahl pathway was present in the genome, which may provide the main carbon source for this bacterium. Genes encoding proteins that play a role in electron transport and energy production, including those encoding F-type ATPase, cytochrome bc1 complex, cytochrome oxidase, and NADH dehydrogenase, were identified. Remarkably, the genome possessed both nitrate reduction and sulfate reduction pathways, as indicated by the presence of the *narGH*, *norBC*, *dsrAB*, and *aprAB* genes. The presence of diverse signal transduction genes, such as *phoRBPA* and *cheWVYA*, supported the tightly regulated metabolic activities. Moreover, a large number of metal ion transporters were present, which are involved in metal efflux and uptake. Compared with the seven close relatives with complete genomes, Nitrospirae bacterium sp. nov displayed a generally similar functional inventory, which included a number of genes related to glycolysis/gluconeogenesis, oxidative phosphorylation, carbon fixation in prokaryotes, dissimilatory nitrate reduction, dissimilatory sulfate reduction, metal ion transport, and signal transduction (Additional file 1: Table S3).

There were 287 proteins with PTMs in the genome of Nitrospirae bacterium sp. nov. The selected proteins with PTMs are highlighted in red in Fig. 4. Some extensively modified proteins included regulators such as PhoR, which regulates PhoBPA to sense phosphate and iron [10]; VicR, which senses osmotic stress [11]; PilR, which is a transcriptional regulator for pilin and other genes required for Fe (III) reduction [12]; and methyl-accepting chemotaxis protein (MCP) and CheW, which are involved in chemotaxis [13]. The F-type ATPase was also found to have unique PTMs. Proteins involved in adenosine-5′-phosphosulfate (APS) and sulfite reduction were modified with PTMs. In addition, transporters such as PstS, which is responsible for phosphate uptake [14], and MlaD, which is responsible for phospholipid transport [15], had PTMs.

## Discussion

We integrated metagenomics and metaproteomics to illustrate protein PTM events in a hydrothermal vent microbial community. The high resolution of the MS system and the protein fractionation allowed an accurate identification, whereas the unexpected high diversity of microbes in the SMAR may have limited the number of detected proteins and modifications. Methylation was one of the dominant PTM events, whereas phosphorylation was among the rare types. In prokaryotes, methyl groups can be added to the carboxyl groups of proteins. Quite few studies have focused on the molecular functions of protein methylation in the microbial world. It has been evidenced that changes in the methylation levels of the chemotaxis signaling proteins correlate with the ability of microbes to response to chemoeffectors [6, 16]. In *Agrobacterium tumefaciens*, methylation of the electron transfer flavoprotein (ETFß) diminished the ability of this enzyme to mediate electron transferring from various dehydrogenases [17]. In the hyperthermophilic archaea, *Sulfolobus islandicus*, the helicase activity of mini-chromosome maintenance (MCM) is enhanced at high temperatures (over 70 °C) by lysine methylation [18]. Collectively, it seems that protein methylation in microbes is involved in signal transduction, energy metabolism, and protein stabilization under high temperatures. These functions and the prevalence of methylation in our metaproteomes led us to assume that methylation would be important for microbial survival under the extreme conditions of the hydrothermal vent. More evidence supporting the role of PTMs in microbial adaptation to the vent environment can be provided by the correlation between PTM types and functional categories, such as the enrichment of hydroxylation for inorganic ion transport and metabolism. Notably, Marlow et al. observed that methylation and hydroxylation were popular PTMs affiliated with orthologs of McrA, a critical enzyme in the reverse methanogenesis pathway, suggesting that the two PTM types may be involved in enzyme regulation in the deep-sea methane seep (775 m depth) [9]. By contrast, the low relative abundance of phosphorylation was not expected because phosphorylation is an important signal transduction mechanism that occurs in prokaryotic organisms [6, 7]. Here, we found that phosphorylation was majorly associated with translation, ribosomal structure, and biosynthesis, suggesting different strategies adopted by the vent microbiome. Moreover, we found that the PTM profile was dictated by taxonomy, whereas the PTMs of orthologous protein differed among microbes, indicating the divergence of PTM patterns underlying the metabolic distinction of closely related microbes.

The present study also demonstrates that the integration of genome binning and proteomics is a good way to identify PTMs in unculturable bacterial species of interest. Members of the Nitrospirae phylum have been reported to inhabit a number of environments, such as acid mine biofilms [8], pond sediments [19], hot springs [20], and lakes [21]. Comparisons in the present study revealed the generally conserved lifestyles of most of the Nitrospirae bacteria with genome sequences. For example, the presence of the Wood-Ljungdahl pathway suggests autotrophic and strictly anaerobic respiration. However, Li et al. proposed that the divergence of PTMs in Nitrospirae may contribute to the phenotypic diversity because the *Leptospirillum* group II dominating AMD biofilms exhibits substantial ecological differentiation [8]. Consistently, PTMs in Nitrospirae bacterium sp. nov in the present study displayed different patterns from those in *Leptospirillum*. In particular, the PTMs of regulators, including PhoR, VicR, and PilR, as well as transporters including PstS and MlaD, suggest an important role of PTMs in metal ion metabolism and resistance, which may facilitate adaptation to the vent area. The detailed functions of PTMs in these novel genes deserve further characterization. In addition, because proteins from different phyla may have quite similar sequences, we cannot be sure that all the protein sequences mapped to Nitrospirae bacterium sp. nov exclusively belong to this organism, and this is would be one of the challenges faced by integrative analysis of metaproteomics and metagenomics.

## Conclusions

PTMs of unique proteins that play a role in energy metabolism, signal transduction, and inorganic ion transport would be an important strategy for microbial adaptation to the vent environment. PTMs in the deep sea are highly diverse and divergent, thus highlighting the need for broader investigations to elucidate their functions.

## Methods

### Sampling

The samples were collected in our 2012 (August) cruise to SMAR (13.35° W, 15.16 °S, and 2500 m in depth) by “Dayang Yihao,” by a conductivity, temperature, and depth (CTD) rosette attached to a remotely operated vehicle (ROV). Hydrothermal activity was confirmed by methane and temperature anomalies with portable miniature autonomous plume recorders attached to a towed deep-sea instrument. The block samples (each of ~1 kg) were collected from the plume wall. After collection, they were immediately transferred to the laboratory with dry ice, followed by frozen in liquid nitrogen and stored at −80 °C until use. Three samples collected decimeters apart were used in the present study: one for the metagenomic sequencing, protein database construction, and genome binning and two for the metaproteomic and PTM analyses.

### DNA extraction and Illumina sequencing

The technique used for DNA extraction has been described in our previous work [22, 23]. Briefly, the hydrothermal plume samples maintained in DNA extraction buffer were homogenized using a sterilized mortar. Subsequently, 50 μl of lysozyme (100 mg/μl) was added to the samples, followed by 400 μl of 20 % SDS and 40 μl of proteinase K (10 μg/μl). The total nucleic acid was then extracted and purified using the AllPrep DNA/RNA Mini Kit (Qiagen, Hilden, Germany). Finally, ~200 ng of DNA was subjected to an Illumina Hiseq 2000 platform (PE500 library) at the Shanghai South Genomics Center (Shanghai, China).

### Metagenome assembly

Illumina reads were subjected to quality control using the next-generation sequencing (NGS) QC toolkit [24] before being assembled using SPAdes Genome Assembler 3.6.1 [25] on a local server. The specified K values 21, 31, 41, 51, 61, 71, and 81 were used under the “--careful” and “--pe” options. ORFs were predicted using Prodigal [26] on a local server, while single procedure and gff output formats were used. The HMMs of conserved single-copy proteins were extracted by searching against a local database.

### Protein extraction and digestion

Protein extraction was performed following the processes described in previous metaproteomic studies [27, 28]. After Coomassie brilliant blue staining, each lane was cut into eight fractions and subjected to in-gel digestion according to the protocol described by Shevchenko et al. [29]. Briefly, the gel fractions were cut into small pieces and placed in Eppendorf tubes. Next, 500 μl of 100 mM ammonium bicarbonate/acetonitrile (1:1, vol/vol) was added to each tube and incubated with occasional vortexing for 300 min. Then, 500 μl of acetonitrile was added to the sample, followed by incubation at room temperature for 30 min. The acetonitrile was then removed, followed by the addition of dithiothreitol solution and incubation at 56 °C for 45 min. The dithiothreitol solution was then removed, and iodoacetamide solution was added followed by incubation in the dark for 30 min. The gel pieces were shrunk with acetonitrile prior to the removal of all liquid. Finally, trypsin buffer was added to cover the dry gel pieces, and the gel was incubated at 4 °C for 30 min before being incubated at 37 °C overnight. The peptides were extracted from the gel slides, desalted, and dried in a speed-vac.

### LC-MS/MS measurements

The dried fractions were re-constituted by 0.1 % formic acid and further analyzed by a LC-Orbitrap Elite mass spectrometer (Thermo Scientific) following our former methods [30]. Briefly, the peptides were fractionated in a 90-min gradient by an Easy-nLC (Thermo Fisher, Bremen, Germany) using a C18 capillary column (Michrom BioResources, CA). The eluted peptides were first scanned in the mass spectrometer with the mass range of 350–1800 m/z and a resolution of 60,000. The top 15 high intensity ions with a minimum threshold of 500 were selected for the downstream fragmentation by using higher-energy collisional dissociation (HCD). The dynamic exclusion with an isolation width of 2.0 m/z and exclusion time of 30 s was adopted. We used 35 % of normalized collision energy and 0.25 activation Q in the HCD analysis.

### Protein and PTM identification

The protein database comprising all ORFs from the abovementioned metagenome was constructed using database maintenance in Mascot (version 2.3.02). The protein identification was performed in reference to the methods described in our previous studies [30]. The MS raw files were processed with Proteome Discoverer 1.0 (Thermo Fisher Scientific) to generate Mascot generic files (*mgf*) of the HCD data. The normalized *mgf* files were submitted to Mascot to search the protein database. The following parameters were used for protein identification: tolerance for parent peptides and fragment ions 5 ppm and 0.3 Da, respectively, and three missed cleavages. All searches were performed with “decoy” sequences. The false discovery rates (FDR) were thus calculated and maintained under 0.1 %. The following settings were used for PTM identification: 5 ppm and 0.02 Da for parent peptides and fragment ions, respectively; up to three missed cleavages; acetylation (N-term; lysine), deamination (glutamine, asparagine, and arginine), hydroxylation (asparagine and lysine), methylation (C-term, aspartic acid, and glutamic acid), nitrosylation (tryptophan and tyrosine), oxidation (methionine, histidine, and tryptophan), and phosphorylation (serine, threonine, and tyrosine) were searched dynamically. PTMs were localized based on the DeltaP score, the score between the two best alternative modification site assignments, as described in previous studies [8, 31]. Taxonomic affinity of proteins of interest was determined by searching against the NCBI-Nr database with blastp (e-value <1e−07) on a local server followed by MEGAN analysis [32].

### Genome binning and validation

Genome binning was performed according to the steps described by Albertsen et al. [33] and in our previous studies [23, 34]. The contigs belonging to different taxa were separated based on the genome coverage, GC content, tetranucleotide frequency, and taxonomic information. The taxonomic information for the contigs was obtained by searching against the NCBI-Nr database with blastp (e-value <1e−07) using a set of conserved single-copy protein-encoding genes as queries, followed by importation of the blast results into MEGAN 5.0 [32]. To exclude potential contig contamination from our genome bins, the extracted contigs could be checked by searching a local database consisting of reported genomes belonging to the same phyla. Here, we constructed a small database comprising the ORFs of seven previously reported complete Nitrospirae genomes [21, 35–37]. The ORFs in the genome bin of the present study were searched against this database with blastp (e-value <1e−07). To further assess the completeness and purity of the genome bin, single-copy protein-coding genes were also compared. In this way, we could determine whether the duplication of single-copy protein-coding genes was caused by contig contamination or incorrect hybridization.

### Genomic analysis

Genomic analysis was performed according to the steps documented in our previous studies [23, 33]. Briefly, the ORFs in the extracted genome were predicted using Prodigal [26] using a local server. The ORFs were annotated by searching the KEGG [38] and COG [39]. Metabolic pathways were revealed using online tools in KEGG Mapper (http://www.genome.jp/kegg/mapper.html).

### Phylogenetic analysis

Phylogenetic organization of the Nitrospirae bacterium sp. nov strain and closely related Nitrospirae strains were visualized based on 16S rRNA sequences (~1400 bp). The reference sequences were retrieved from the NCBI database. Alignment was made by ClustalW implemented by Molecular Evolutionary Genetics Analysis (MEGA, version 6.05) [40], and then, a maximum likelihood tree was constructed. Phylogeny was also investigated based on conserved single-copy genes, which were widely used in microbiome studies [41–43]. AMPHORA [44] was used to predict conserved single-copy genes from the genome of Nitrospirae bacterium sp. nov and all the available Nitrospirae genomes (draft and complete genomes) in the NCBI database. Protein sequences corresponding to twelve single-copy genes (*tsf*, *rpsS*, *rpsJ*, *rpsE*, *rpmA*, *rplT*, *rplS*, *rplF*, *rplE*, *rplC*, *rplB*, and *pgk*) that were present in all the involved genomes were aligned by ClustalW. The aligned protein sequences were then concatenated using an in-house script and imported to Mega to construct a ML tree based on the Jones-Taylor-Thornton (JTT) substitution model. The bootstrap values were calculated with 500 replicates.

## Additional files

Additional file 1: Table S1. Features of the assembled metagenomes and binned draft genomes. **Table S2.** Conserved single-copy protein-coding genes for the estimation of genome completeness. The numbers of the 139 single copy genes in Nitrospirae bacterium sp. nov were compared with that in closely related genomes. **Table S3.** Numbers of genes involved in carbohydrate metabolism, nitrogen metabolism, and sulfur metabolism in Nitrospirae bacterium sp. nov and the reference genomes. **Figure S1.** Work flow of the present study. Three samples were collected decimeters apart: one for the metagenomic sequencing, protein database construction, and genome binning and two for the metaproteomic and PTM analyses. **Figure S2.** A Venn diagram showing the overlaps between identified proteins (a) and PTMs (b) in the two metaproteomic samples. **Figure S3.** Alignments of partial F-type-ATPase protein sequences to show the PTM sites. **Figure S4.** Phylogenetic organization of the Nitrospirae bacterium sp. nov strain and closely related Nitrospirae strains based on 16S rRNA sequences (~1400 bp). **Figure S5.** Phylogenetic organization of the Nitrospirae bacterium sp. nov strain and Nitrospirae strains based on concatenated single-copy genes. (DOC 1298 kb) Additional file 2: List of PTMs. (XLSX 696 kb)

## Abbreviations

PTM: Post-translational modification
AMD: Acid mine drainage
Mcr: Methyl-coenzyme M reductase
SMAR: South Mid Atlantic Ridge
HMMs: Hidden Markov models
COGs: Clusters of Orthologous Groups
KEGG: Kyoto Encyclopedia of Genes and Genomes
MEGAN: MEtaGenome ANalyzer
MCP: Methyl-accepting chemotaxis protein
APS: Adenosine-5′-phosphosulfate
MCM: Mini-chromosome maintenance
CTD: Conductivity, temperature, and depth
ROV: Remotely operated vehicle
NGS: Next-generation sequencing
HCD: Higher-energy collisional dissociation
mgf: Mascot generic files
FDR: False discovery rates
MEGA: Molecular Evolutionary Genetics Analysis
JTT: Jones-Taylor-Thornton
