# Supplementary material for: Post-translational modifications are enriched within protein functional groups important to bacterial adaptation within a deep-sea hydrothermal vent environment
Source: Microbiome. 2016 Sep 6;4(1):49. doi: 10.1186/s40168-016-0194-x (PMC5012046; doi:10.1186/s40168-016-0194-x)
Supplement: Additional file 1: Table S1. — Features of the assembled metagenomes and binned draft genomes. Table S2. Conserved single-copy protein-coding genes for the estimation of genome completeness. The numbers of the 139 single copy genes in Nitrospirae bacterium sp. nov were compared with that in closely related genomes. Table S3. Numbers of genes involved in carbohydrate metabolism, nitrogen metabolism, and sulfur metabolism in Nitrospirae bacterium sp. nov and the reference genomes. Figure S1. Work flow of the present study. Three samples were collected decimeters apart: one for the metagenomic sequencing, protein database construction, and genome binning and two for the metaproteomic and PTM analyses. Figure S2. A Venn diagram showing the overlaps between identified proteins (a) and PTMs (b) in the two metaproteomic samples. Figure S3. Alignments of partial F-type-ATPase protein sequences to show the PTM sites. Figure S4. Phylogenetic organization of the Nitrospirae bacterium sp. nov strain and closely related Nitrospirae strains based on 16S rRNA sequences (~1400 bp). Figure S5. Phylogenetic organization of the Nitrospirae bacterium sp. nov strain and Nitrospirae strains based on concatenated single-copy genes. (DOC 1298 kb) [file 40168_2016_194_MOESM1_ESM.doc]

**Additional file 1: Table S1.** Features of the assembled metagenomes and binned draft genomes.

| **Features** | **Assembled metagenome** | **Nitrospirae bacterium sp. nov genome** |
| --- | --- | --- |
| Size (Mbp) | 153,137 | 1.9 |
| Genome recovery (%) | - | ~85.6 |
| No. of ribosomal RNA | 197 | 4 |
| No. of conserved single-copy genes | 3,577 | 129 |
| No. of contigs | 24,099 | 230 |
| N50 of contigs | 10,361 | 11,200 |
| N90 of contigs | 2,513 | 3,814 |
| No. of ORFs | 171,515 | 1,994 |
| GC content (%) | 48.1 | 47.7 |

**Additional file 1: Table S2. Conserved single-copy protein-coding genes for the estimation of genome completeness. The completeness of each population bin was evaluated using a suite of Hidden Markov Models (HMM) including 139 proteins conserved in 95% of all sequenced bacterial genomes. The numbers of the 139 single copy genes in Nitrospirae bacterium sp. nov were compared with that in closely related genomes. 1, Nitrospirae bacterium sp. nov; 2, Candidatus *Nitrospira defluvii*; 3, Candidatus *Magnetobacterium casensis* strain MYR-1; 4, *Leptospirillum ferriphilum* ML-04; 5, *Leptospirillum ferriphilum* YSK; 6, *Leptospirillum ferrooxidans* C2-3; 7, *Nitrospira moscoviensis* strain NSP M-1; 8, *Thermodesulfovibrio yellowstonii* DSM 11347.**

| **HMM name** | **Gene name** | **Genomes** | | | | | | | |
| --- | --- | --- | --- | --- | --- | --- | --- | --- | --- |
|  |  | **1** | **2** | **3** | **4** | **5** | **6** | **7** | **8** |
| PF03485 | Arg_tRNA_synt_N | 1 | 1 | 2 | 1 | 1 | 1 | 1 | 1 |
| PF03484 | B5 | 1 | 1 | 1 | 1 | 1 | 1 | 1 | 1 |
| PF01121 | CoaE | 1 | 1 | 1 | 1 | 1 | 1 | 1 | 1 |
| PF03772 | Competence | 1 | 1 | 1 | 1 | 1 | 1 | 1 | 1 |
| PF03602 | Cons_hypoth95 | 1 | 1 | 1 | 1 | 1 | 1 | 1 | 1 |
| PF06418 | CTP_synth_N | 1 | 1 | 1 | 1 | 1 | 1 | 1 | 1 |
| PF02224 | Cytidylate_kin | 0 | 1 | 1 | 1 | 1 | 1 | 1 | 1 |
| PF00712 | DNA_pol3_beta | 1 | 1 | 1 | 1 | 1 | 1 | 3 | 1 |
| PF02767 | DNA_pol3_beta_2 | 0 | 1 | 1 | 1 | 1 | 1 | 3 | 1 |
| PF02768 | DNA_pol3_beta_3 | 0 | 1 | 1 | 1 | 1 | 1 | 3 | 1 |
| PF00035 | dsrm | 1 | 1 | 1 | 2 | 2 | 2 | 1 | 0 |
| PF00889 | EF_TS | 2 | 2 | 2 | 2 | 2 | 2 | 2 | 2 |
| PF01176 | eIF_1a | 1 | 1 | 1 | 1 | 1 | 1 | 1 | 1 |
| PF00113 | Enolase_C | 1 | 1 | 1 | 1 | 1 | 1 | 1 | 1 |
| PF03952 | Enolase_N | 1 | 1 | 1 | 1 | 1 | 1 | 1 | 1 |
| PF06574 | FAD_syn | 1 | 1 | 0 | 1 | 1 | 1 | 1 | 1 |
| PF03147 | FDX_ACB | 1 | 0 | 1 | 0 | 0 | 0 | 0 | 1 |
| PF01687 | Flavokinase | 1 | 1 | 0 | 1 | 1 | 1 | 1 | 1 |
| PF02938 | GAD | 0 | 1 | 1 | 1 | 1 | 1 | 1 | 1 |
| PF02527 | GidB | 1 | 1 | 1 | 1 | 1 | 1 | 0 | 1 |
| PF00958 | GMP_synt_C | 1 | 1 | 1 | 1 | 1 | 1 | 1 | 1 |
| PF01025 | GrpE | 2 | 1 | 2 | 1 | 1 | 1 | 1 | 1 |
| PF01018 | GTP1_OBG | 1 | 1 | 1 | 1 | 1 | 1 | 1 | 1 |
| PF11987 | IF_2 | 1 | 1 | 1 | 1 | 1 | 1 | 1 | 1 |
| PF04760 | IF2_N | 2 | 2 | 2 | 2 | 2 | 2 | 2 | 1 |
| PF00707 | IF3_C | 1 | 1 | 1 | 1 | 1 | 1 | 1 | 1 |
| PF05198 | IF3_N | 1 | 0 | 1 | 1 | 1 | 0 | 1 | 0 |
| PF01715 | IPPT | 1 | 1 | 1 | 1 | 1 | 1 | 1 | 1 |
| PF06421 | LepA_C | 1 | 1 | 1 | 1 | 1 | 1 | 1 | 1 |
| PF01795 | Methyltransf_5 | 1 | 1 | 1 | 1 | 1 | 1 | 1 | 1 |
| PF02873 | MurB_C | 0 | 1 | 1 | 1 | 1 | 1 | 1 | 1 |
| PF08529 | NusA_N | 1 | 1 | 1 | 1 | 1 | 1 | 1 | 1 |
| PF02410 | Oligomerisation | 2 | 1 | 1 | 1 | 1 | 1 | 1 | 2 |
| PF01195 | Pept_tRNA_hydro | 1 | 1 | 1 | 1 | 1 | 1 | 1 | 1 |
| PF01252 | Peptidase_A8 | 1 | 1 | 1 | 1 | 1 | 1 | 1 | 1 |
| PF00162 | PGK | 1 | 1 | 1 | 1 | 1 | 1 | 1 | 1 |
| PF02912 | Phe_tRNA_synt_N | 1 | 0 | 1 | 0 | 0 | 0 | 0 | 1 |
| PF03726 | PNPase | 1 | 1 | 1 | 1 | 1 | 1 | 1 | 1 |
| PF01416 | PseudoU_synth_1 | 2 | 2 | 2 | 2 | 2 | 2 | 2 | 2 |
| PF02033 | RBFA | 1 | 1 | 1 | 1 | 1 | 1 | 1 | 1 |
| PF00154 | RecA | 1 | 1 | 1 | 1 | 1 | 1 | 1 | 1 |
| PF02132 | RecR | 1 | 1 | 1 | 1 | 1 | 0 | 1 | 1 |
| PF00825 | Ribonuclease_P | 0 | 1 | 1 | 0 | 0 | 1 | 1 | 0 |
| PF00687 | Ribosomal_L1 | 1 | 1 | 1 | 1 | 1 | 1 | 1 | 1 |
| PF00466 | Ribosomal_L10 | 0 | 1 | 1 | 1 | 1 | 1 | 1 | 1 |
| PF00298 | Ribosomal_L11 | 1 | 1 | 1 | 1 | 1 | 1 | 1 | 1 |
| PF03946 | Ribosomal_L11_N | 1 | 1 | 1 | 1 | 1 | 1 | 1 | 1 |
| PF00542 | Ribosomal_L12 | 0 | 1 | 1 | 1 | 1 | 1 | 1 | 1 |
| PF00572 | Ribosomal_L13 | 0 | 1 | 1 | 1 | 1 | 1 | 1 | 2 |
| PF00238 | Ribosomal_L14 | 1 | 1 | 1 | 0 | 0 | 1 | 1 | 1 |
| PF00252 | Ribosomal_L16 | 1 | 1 | 1 | 0 | 0 | 1 | 1 | 1 |
| PF01196 | Ribosomal_L17 | 1 | 1 | 1 | 1 | 1 | 1 | 1 | 1 |
| PF00828 | Ribosomal_L18e | 1 | 1 | 1 | 1 | 1 | 1 | 1 | 1 |
| PF00861 | Ribosomal_L18p | 1 | 1 | 1 | 1 | 1 | 1 | 1 | 1 |
| PF01245 | Ribosomal_L19 | 1 | 1 | 1 | 1 | 1 | 1 | 1 | 1 |
| PF00181 | Ribosomal_L2 | 1 | 1 | 1 | 1 | 1 | 1 | 1 | 1 |
| PF03947 | Ribosomal_L2_C | 1 | 1 | 1 | 1 | 1 | 1 | 1 | 1 |
| PF00453 | Ribosomal_L20 | 1 | 1 | 1 | 1 | 1 | 1 | 1 | 1 |
| PF00829 | Ribosomal_L21p | 1 | 1 | 1 | 1 | 1 | 1 | 1 | 1 |
| PF00237 | Ribosomal_L22 | 1 | 1 | 1 | 1 | 1 | 1 | 1 | 1 |
| PF00276 | Ribosomal_L23 | 1 | 1 | 1 | 1 | 1 | 1 | 1 | 1 |
| PF01016 | Ribosomal_L27 | 1 | 1 | 1 | 1 | 1 | 1 | 1 | 1 |
| PF00830 | Ribosomal_L28 | 1 | 1 | 1 | 0 | 0 | 0 | 1 | 1 |
| PF00831 | Ribosomal_L29 | 1 | 1 | 1 | 1 | 1 | 1 | 1 | 1 |
| PF00297 | Ribosomal_L3 | 1 | 1 | 1 | 1 | 1 | 1 | 1 | 1 |
| PF01783 | Ribosomal_L32p | 1 | 1 | 1 | 1 | 1 | 1 | 1 | 1 |
| PF01632 | Ribosomal_L35p | 1 | 1 | 1 | 1 | 1 | 1 | 1 | 1 |
| PF00573 | Ribosomal_L4 | 1 | 1 | 1 | 1 | 1 | 1 | 1 | 1 |
| PF00281 | Ribosomal_L5 | 1 | 1 | 1 | 1 | 1 | 1 | 1 | 1 |
| PF00673 | Ribosomal_L5_C | 1 | 1 | 1 | 1 | 1 | 1 | 1 | 1 |
| PF00347 | Ribosomal_L6 | 2 | 2 | 2 | 2 | 2 | 2 | 2 | 2 |
| PF03948 | Ribosomal_L9_C | 1 | 1 | 1 | 1 | 1 | 1 | 1 | 1 |
| PF01281 | Ribosomal_L9_N | 1 | 1 | 1 | 1 | 1 | 1 | 1 | 1 |
| PF00338 | Ribosomal_S10 | 1 | 1 | 1 | 1 | 1 | 1 | 1 | 1 |
| PF00411 | Ribosomal_S11 | 1 | 1 | 1 | 0 | 0 | 1 | 1 | 1 |
| PF00164 | Ribosomal_S12 | 1 | 1 | 1 | 1 | 1 | 1 | 1 | 1 |
| PF00416 | Ribosomal_S13 | 1 | 1 | 1 | 1 | 1 | 1 | 1 | 1 |
| PF00312 | Ribosomal_S15 | 1 | 1 | 0 | 1 | 1 | 1 | 1 | 1 |
| PF00886 | Ribosomal_S16 | 1 | 1 | 1 | 1 | 1 | 1 | 1 | 1 |
| PF00366 | Ribosomal_S17 | 1 | 1 | 1 | 0 | 0 | 1 | 1 | 1 |
| PF01084 | Ribosomal_S18 | 1 | 1 | 1 | 1 | 1 | 1 | 1 | 1 |
| PF00203 | Ribosomal_S19 | 1 | 1 | 1 | 1 | 1 | 1 | 1 | 1 |
| PF00318 | Ribosomal_S2 | 1 | 1 | 1 | 1 | 1 | 1 | 1 | 1 |
| PF01649 | Ribosomal_S20p | 0 | 1 | 1 | 1 | 1 | 1 | 1 | 1 |
| PF00189 | Ribosomal_S3_C | 1 | 1 | 1 | 1 | 1 | 1 | 1 | 1 |
| PF00163 | Ribosomal_S4 | 1 | 1 | 1 | 1 | 1 | 1 | 1 | 1 |
| PF00333 | Ribosomal_S5 | 1 | 1 | 1 | 1 | 1 | 1 | 1 | 1 |
| PF03719 | Ribosomal_S5_C | 1 | 1 | 1 | 1 | 1 | 1 | 1 | 1 |
| PF01250 | Ribosomal_S6 | 1 | 1 | 1 | 1 | 1 | 1 | 1 | 1 |
| PF00177 | Ribosomal_S7 | 1 | 1 | 1 | 1 | 1 | 1 | 1 | 1 |
| PF00410 | Ribosomal_S8 | 1 | 1 | 1 | 1 | 1 | 1 | 1 | 1 |
| PF00380 | Ribosomal_S9 | 0 | 1 | 1 | 1 | 1 | 1 | 1 | 2 |
| PF01782 | RimM | 1 | 1 | 0 | 0 | 0 | 0 | 1 | 0 |
| PF01000 | RNA_pol_A_bac | 1 | 1 | 1 | 1 | 1 | 1 | 1 | 1 |
| PF03118 | RNA_pol_A_CTD | 1 | 1 | 1 | 1 | 1 | 1 | 1 | 1 |
| PF01193 | RNA_pol_L | 1 | 1 | 1 | 1 | 1 | 1 | 1 | 1 |
| PF04997 | RNA_pol_Rpb1_1 | 1 | 1 | 1 | 2 | 2 | 2 | 1 | 1 |
| PF00623 | RNA_pol_Rpb1_2 | 1 | 1 | 1 | 1 | 1 | 1 | 1 | 1 |
| PF04983 | RNA_pol_Rpb1_3 | 1 | 1 | 1 | 1 | 1 | 1 | 1 | 1 |
| PF05000 | RNA_pol_Rpb1_4 | 1 | 1 | 1 | 1 | 1 | 1 | 1 | 1 |
| PF04998 | RNA_pol_Rpb1_5 | 1 | 1 | 1 | 1 | 1 | 1 | 1 | 1 |
| PF04563 | RNA_pol_Rpb2_1 | 0 | 1 | 1 | 1 | 1 | 1 | 1 | 1 |
| PF04561 | RNA_pol_Rpb2_2 | 0 | 2 | 2 | 2 | 2 | 2 | 2 | 2 |
| PF04565 | RNA_pol_Rpb2_3 | 0 | 1 | 1 | 1 | 1 | 1 | 1 | 1 |
| PF10385 | RNA_pol_Rpb2_45 | 0 | 1 | 1 | 1 | 1 | 1 | 1 | 1 |
| PF00562 | RNA_pol_Rpb2_6 | 1 | 1 | 1 | 1 | 1 | 1 | 1 | 1 |
| PF04560 | RNA_pol_Rpb2_7 | 1 | 1 | 1 | 1 | 1 | 1 | 1 | 1 |
| PF01765 | RRF | 1 | 1 | 1 | 1 | 1 | 1 | 1 | 1 |
| PF07499 | RuvA_C | 0 | 1 | 1 | 0 | 0 | 0 | 1 | 1 |
| PF01330 | RuvA_N | 0 | 1 | 1 | 0 | 0 | 1 | 1 | 1 |
| PF05491 | RuvB_C | 0 | 1 | 1 | 1 | 1 | 1 | 1 | 1 |
| PF02773 | S_AdoMet_synt_C | 2 | 1 | 0 | 1 | 1 | 1 | 1 | 1 |
| PF02772 | S_AdoMet_synt_M | 1 | 1 | 0 | 1 | 1 | 1 | 1 | 1 |
| PF00584 | SecE | 1 | 1 | 1 | 1 | 1 | 1 | 1 | 1 |
| PF03840 | SecG | 1 | 1 | 1 | 1 | 1 | 1 | 1 | 1 |
| PF00344 | SecY | 1 | 1 | 1 | 1 | 1 | 1 | 1 | 1 |
| PF02403 | Seryl_tRNA_N | 1 | 1 | 1 | 1 | 1 | 1 | 1 | 1 |
| PF01668 | SmpB | 1 | 1 | 0 | 1 | 1 | 1 | 1 | 1 |
| PF02978 | SRP_SPB | 1 | 1 | 1 | 1 | 1 | 1 | 1 | 1 |
| PF00763 | THF_DHG_CYH | 2 | 1 | 2 | 1 | 1 | 1 | 1 | 1 |
| PF02882 | [THF_DHG_CYH_C](http://pfam.xfam.org/family/PF02882) | 2 | 1 | 2 | 1 | 1 | 1 | 1 | 1 |
| PF00121 | TIM | 1 | 1 | 1 | 1 | 1 | 1 | 1 | 1 |
| PF08275 | Toprim_N | 0 | 1 | 0 | 1 | 1 | 1 | 2 | 1 |
| PF03461 | TRCF | 1 | 1 | 1 | 1 | 1 | 1 | 1 | 1 |
| PF05698 | Trigger_C | 1 | 1 | 1 | 0 | 0 | 0 | 1 | 1 |
| PF05697 | Trigger_N | 1 | 1 | 1 | 1 | 1 | 1 | 1 | 1 |
| PF01746 | tRNA_m1G_MT | 1 | 1 | 1 | 1 | 1 | 1 | 1 | 1 |
| PF00750 | tRNA_synt_1d | 1 | 1 | 3 | 1 | 1 | 1 | 1 | 1 |
| PF01409 | Trna_synt_2d | 1 | 1 | 1 | 1 | 1 | 1 | 1 | 1 |
| PF01509 | TruB_N | 1 | 1 | 1 | 1 | 1 | 1 | 1 | 1 |
| PF00627 | UBA | 1 | 1 | 1 | 1 | 1 | 1 | 1 | 1 |
| PF02130 | UPF0054 | 1 | 1 | 1 | 1 | 1 | 1 | 1 | 1 |
| PF02367 | UPF0079 | 1 | 1 | 1 | 1 | 1 | 1 | 1 | 1 |
| PF03652 | UPF0081 | 0 | 1 | 0 | 1 | 1 | 1 | 1 | 0 |
| PF12344 | UvrB | 1 | 1 | 1 | 1 | 1 | 1 | 1 | 1 |
| PF08459 | UvrC_HhH_N | 1 | 1 | 1 | 1 | 1 | 1 | 1 | 1 |
| PF10458 | Val_tRNA_synt_C | 1 | 1 | 0 | 1 | 1 | 1 | 1 | 1 |
| PF06071 | YchF_GTPase_C | 1 | 1 | 1 | 1 | 1 | 1 | 1 | 1 |
| PF06689 | Zf_C4_ClpX | 1 | 1 | 1 | 1 | 1 | 1 | 1 | 1 |

**Additional file 1: Table S3. Numbers of genes involved in carbohydrate metabolism, nitrogen metabolism and sulfur metabolism in 1, Nitrospirae bacterium sp. nov; 2, Candidatus *Nitrospira defluvii*; 3, Candidatus *Magnetobacterium casensi*s strain MYR-1; 4, *Leptospirillum ferriphilum* ML-04; 5, *Leptospirillum ferriphilum* YSK; 6, *Leptospirillum ferrooxidans* C2-3; 7, *Nitrospira moscoviensis* strain NSP M-1; 8, *Thermodesulfovibrio yellowstonii* DSM 11347. The gene annotation was based on the KEGG database.**

| **Function** | **Genomes** | | | | | | | |
| --- | --- | --- | --- | --- | --- | --- | --- | --- |
|  | **1** | **2** | **3** | **4** | **5** | **6** | **7** | **8** |
| [**Glycolysis/Gluconeogenesis**](http://www.genome.jp/kegg-bin/show_pathway?142573660423596/map00010.coords+reference) | 23 | 25 | 17 | 21 | 18 | 17 | 28 | 20 |
| **Oxidative phosphorylation** | 24 | 31 | 28 | 29 | 30 | 33 | 29 | 27 |
| **Carbon fixation in prokaryotes** | 6 | 4 | 7 | 4 | 4 | 4 | 4 | 5 |
| **Dissimilatory nitrate reduction** | 2 | 2 | 4 | 1 | 1 | 1 | 2 | 2 |
| **Dissimilatory sulfate reduction** | 3 | 0 | 3 | 2 | 2 | 2 | 0 | 3 |
| **ABC transporters** |  | | | | | | | |
| Mineral and organic ion transporters | 5 | 15 | 1 | 1 | 2 | 2 | 6 | 2 |
| Phosphate and amino acid transporters | 6 | 7 | 8 | 4 | 4 | 4 | 4 | 13 |
| ABC-2 transporters | 6 | 10 | 10 | 8 | 8 | 8 | 7 | 8 |
| Cation, Iron-siderophore transporters | 8 | 4 | 11 | 0 | 0 | 0 | 3 | 12 |
| **Two-component system** |  | | | | | | | |
| OmpR family | 7 | 7 | 7 | 4 | 5 | 4 | 5 | 7 |
| NtrC family | 7 | 12 | 9 | 5 | 5 | 6 | 5 | 8 |
| Chemotaxis family | 10 | 11 | 7 | 7 | 7 | 6 | 10 | 9 |

**Additional file 1: Figure S1.** Work flow of the present study. Three samples were collected decimeters apart: one for the metagenomic sequencing, protein database construction and genome binning, and two for the metaproteomic and PTM analyses. The samples were collected in August, 2012 cruise to SMAR (13.35°W, 15.16°S and 2,500 m in depth).

**Additional file 1: Figure S2.** A Venn diagram showing the overlaps between identified proteins (a) and PTMs (b) in the two metaproteomic samples.

**Additional file 1: Figure S3.** Alignments of partial F-type-ATPase protein sequences to show the PTM sites. The PTM types are highlighted in different colors. Conserved amino acids are indicated by ‘*’. The ATPases 1-3 belonged to Bacteroidia, Gammaproteobacteria and Flavobacteriia, respectively, and ATPases 4-7 showed high affinity to Alphaproteobacteria, according to the results of MEGAN analysis. Oxidation of Met may also result from protein sample preparation process.

**Additional file 1: Figure S4.** Phylogenetic organization of the Nitrospirae bacterium sp. nov strain and closely related Nitrospirae strains based on 16S rRNA sequences (~1400bp). The maximum likelihood method was used to construct the phylogenetic tree. The reference sequences were retrieved from the NCBI database. Bootstrap values based on 500 replications are shown on the nodes. The ‘#’ indicates strains with available complete genome sequences used for genomic comparisons in the present study.

**Additional file 1: Figure S5.** Phylogenetic organization of the Nitrospirae bacterium sp. nov strain and Nitrospirae strains based on concatenated single-copy genes. The maximum likelihood method was used to construct the phylogenetic tree. The reference genomes were retrieved from the NCBI database. Bootstrap values based on 500 replications are shown on the nodes.
